# Effects of Lutein on Hyperosmoticity-Induced Upregulation of IL-6 in Cultured Corneal Epithelial Cells and Its Relevant Signal Pathways

**DOI:** 10.1155/2016/8341439

**Published:** 2016-03-07

**Authors:** Shih-Chun Chao, Chan-Wei Nien, Codrin Iacob, Dan-Ning Hu, Sheng-Chieh Huang, Hung-Yu Lin

**Affiliations:** ^1^Department of Ophthalmology, Show Chwan Memorial Hospital, No. 526, Section 1, Zhongshan Road, Changhua 500, Taiwan; ^2^Institute of Electrical and Computer Engineering, National Chiao Tung University, Hsinchu 30010, Taiwan; ^3^Central Taiwan University of Science and Technology, No. 666, Buzih Road, Beitun District, Taichung 40601, Taiwan; ^4^New York Eye and Ear Infirmary of Mount Sinai, 310 East 14th Street, New York, NY 10003, USA; ^5^Department of Optometry, Yuan Pei University, Hsinchu 30015, Taiwan; ^6^Department of Optometry, Chung Shan Medical University, Taichung 40201, Taiwan

## Abstract

Dry eye is a common disorder characterized by deficiency of tear. Hyperosmoticity of tear stimulates inflammation and damage of ocular surface tissues and plays an essential role in the pathogenesis of dry eye. Cultured human corneal epithelial (CE) cells were used for the study of effects of lutein and hyperosmoticity on the secretion of IL-6 by CE cells. Cell viability of CE cells was not affected by lutein at 1–10 *μ*M as determined by MTT assay. Hyperosmoticity significantly elevated the secretion of IL-6 by CE cells as measured by ELISA analysis. The constitutive secretion of IL-6 was not affected by lutein. Lutein significantly and dose-dependently inhibited hyperosmoticity-induced secretion of IL-6. Phosphorylated- (p)- p38 MAPK, p-JNK levels in cell lysates and NF-*κ*B levels in cell nuclear extracts were increased by being exposed to hyperosmotic medium. JNK, p38, and NF-*κ*B inhibitors decreased hyperosmoticity-induced secretion of IL-6. Lutein significantly inhibited hyperosmoticity-induced elevation of NF-*κ*B, p38, and p-JNK levels. We demonstrated that lutein inhibited hyperosmoticity-induced secretion of IL-6 in CE cells through the deactivation of p38, JNK, and NF-*κ*B pathways. Lutein may be a promising agent to be explored for the treatment of dry eye.

## 1. Introduction

Dry eye is a multifactorial disorder of the ocular surface characterized by symptoms of ocular discomfort and visual disturbance and is associated with reductions in the quality and/or quantity of tears. Symptoms of dry eye include eye irritation, stinging, dryness, eye fatigue, and fluctuating visual disturbances. Dry eye is a common disease and the prevalence of dry eye ranges from 5 to 33% of the adult population. The prevalence of dry eye increases with age and is more common in women. It can lead to significant functional impairment in daily life and affects quality of life and productivity. A substantial economic burden to the patients and society is derived owing to associated health care costs and loss of productivity of affected individuals [1–7].

Deficiency of tear, resulting from the decrease of aqueous tear production or excessive tear evaporation, is the essential pathological changes of dry eye. Hyperosmolarity of tear film caused by deficiency of tear can initiate inflammation and damage of ocular surface and is the main mechanism of the development of dry eye [1, 4, 8].

Inflammation plays an important role in the pathogenesis of dry eye. It has been reported that various proinflammatory cytokines and chemokines levels in the tears or ocular surface tissues are significantly increased in dry eye patients [9–14]. IL-6 is a proinflammatory cytokine and plays an important role in the pathogenesis of autoimmune diseases [15, 16]. IL-6 levels in the tear are significantly increased in dry eye patients [9–12]. IL-6 levels rise in lacrimal and ocular surface tissues in patients with Sjögren syndrome (an autoimmune disorder and an important cause of dry eye) [13, 14]. The expression and production of IL-6 are also elevated in experimental dry eye models [8, 17–19]. It has been reported that hyperosmoticity can stimulate the expression and secretion of IL-6 in various cultured cells [8, 18, 19].

Lutein, a natural bioactive substance that belongs to the xanthophyll class of the carotenoids, is found in dark green leafy vegetables such as kale and spinach. Lutein is naturally present in the eye at a high level. It is a yellow colored pigment that can absorb high energy blue light and protects cells from phototoxicity. Lutein also works as an antioxidant and a free radical scavenger [20–23].

Lutein has been studied for its effects on the prevention and treatment of various ophthalmic diseases in vitro and in vivo, including age-related macular degeneration (AMD) and diabetic retinopathy [24–30]. Epidemiological studies and clinical trials documented that a high level of lutein in the macula is associated with lower incidence of AMD and supplementation of lutein has effects on the prevention and treatment of AMD. Therefore, lutein is widely used as nutrient supplements for the management of AMD and other eye diseases [31–34].

Recently, a number of in vivo and in vitro studies suggested that lutein had an anti-inflammatory effect in experimental animal uveitis models and in cultured cells stimulated by various proinflammatory factors [35–39]. Our previous study documented that lutein inhibited lipopolysaccharide- (LPS-) induced secretion of IL-8 in cultured uveal melanocytes, which suggested that lutein may be a promising agent to be explored for the prevention and treatment of ocular inflammation [35]. Furthermore, blood lutein levels are inversely related to IL-6 levels in normal individuals or patients with various diseases [40–42]; supplementation of lutein decreases IL-6 levels in experimental animals or patients [43–45]. To the best of our knowledge, the effects of lutein on the inflammatory processes of ocular surface have not been reported.

The purpose of the present study was to investigate hyperosmoticity-induced upregulation of IL-6 in cultured human CE cells and its relevant signal pathways.

## 2. Material and Methods

### 2.1. Cell Culture

The human corneal epithelial (CE) cells used in the present study are a SV40-adenovirus-immortalized CE cell line, which was obtained from Dr. Peter Reinach (State University of New York, New York, USA) [8]. Cells were grown in defined K-SFM medium supplemented with 10% fetal bovine serum and 50 *μ*g/mL gentamicin (all from GIBCO, Grand Island, NY, USA) and incubated in a humidified 95% air/5% CO_2_ atmosphere at 37°C [8].

### 2.2. MTT Assay

The effects of lutein on the cell viability of cultured CE were tested by using MTT assay as previously described [8]. For each experiment, cells were seeded into 96-well plates at a density of 5 × 10^3^ cells/well. Lutein (Sigma, St. Louis, MO, USA) was dissolved in DMSO (Sigma) and added to the medium at different levels 24 h later. After incubation for 24 h, MTT (50 *μ*L/well of 1 mg/mL) was added and cells were incubated for another 4 h. DMSO at 100 *μ*L/well was added after the removal of the culture medium. The optical density as the parameter of cell viability was measured at 540 nm with a microplate reader (Multiskan EX, Thermo, Ventana, Finland). All experiments were performed in triplicate.

### 2.3. Effects of Lutein and Hyperosmoticity on the Secretion of IL-6 by CE Cells

For the study of secretion of IL-6 by CE cells, 5 × 10^4^ cells were seeded on 24-well plates. After 24 h, the culture medium was replaced with serum-free isoosmotic or hyperosmotic medium and cultured for 24 h. For the preparation of hyperosmotic medium, sodium chloride was added to the medium to reach a final concentration of 90 mM with hyperosmoticity at 450 mOsM [8]. Osmette Osmometer (Precision System, Natick, MA, USA) was used to measure the osmolarity of the solution [8]. In the study of the effects of lutein, lutein at 0, 1, 3, and 10 *μ*M was added 30 min before exposure to the hyperosmotic medium and then cultured for 24 h. Conditioned culture media from different groups were collected and centrifuged. The supernatants were stored at −80°C. All experiments were performed in triplicate.

### 2.4. Measurement of IL-6 Protein Levels

IL-6 protein levels in the conditioned culture medium were measured by enzyme-linked immunosorbent assay (ELISA) by using the human IL-6 Quantikine ELISA kit (R&D System, Minneapolis, MN, USA) in accordance with the manufacturer's instructions. The optical density of the ELISA samples was measured at 450 and 540 nm using a microplate reader; IL-6 levels (pg/mL) were calculated from a standard curve and expressed as the percentage of the negative controls (cells cultured with isoosmotic medium without lutein). The sensitivity of the assay was 0.7 pg/mL. All experiments were performed in triplicate.

### 2.5. Effects of Lutein and Hyperosmoticity on MAPK and NF-*κ*B Levels in CE Cells

CE cells at a density of 1 × 10^6^ cells/well were seeded on 6-well plates and cultured for 24 h. Then, culture medium was removed and washed and cells were cultured with isoosmotic or hyperosmotic medium with and without lutein (10 *μ*M) as described above. The cultures were washed with cold PBS 60 min later. Cells were collected and centrifuged. The pellets were cultured with cell extraction buffer (Biosource, Camarillo, CA, USA), protease inhibitor cocktail (Sigma), and PMSF (Biosource) for 30 min at 4°C with vortexing at 10 min intervals. Cultures were microcentrifuged at 4°C and the supernatants were collected and stored at −80°C until analysis for mitogen-activated protein kinase (MAPK) levels. For the measurement of nuclear factor-kappa B (NF-*κ*B) levels in cell nucleus, cells were collected, treated with hypotonic buffer (BioSource), and centrifuged. The pellets that contained nuclear fraction were collected, treated with cell extraction buffer (BioSource), vortexed, and centrifuged. The supernatants were stored at −80°C until analysis for NF-*κ*B levels.

### 2.6. Measurement of MAPK and NF-*κ*B Levels

ELISA was used for the measurement of MAPK and NF-*κ*B levels. MAPK levels were measured by using various phosphorylated- (p)- MAPK kits (Biosource). P-p38 MAPK, p-extracellular signal-regulated kinases 1/2 (ERK1/2), and p-c-Jun N-terminal kinase (JNK1/2) kits were used to determine p-p38 MAPK, p-ERK1/2, and p-JNK1/2 levels in cell extracts, respectively. The test was performed according to the protocol provided by the manufacturer and expressed as the percentage of the negative controls (cells cultured with isoosmotic medium without lutein). The sensitivity of these kits was 0.8 U/mL. NF-*κ*B levels in the nuclear portion were measured by NF-*κ*B ELISA kits (Invitrogen) according to the manufacturer's instructions. The levels of NF-*κ*B were expressed as percentages of the negative controls. The sensitivity of this kit was <50 pg/mL. All tests were performed in triplicate.

### 2.7. Effects of MAPK and NF-*κ*B Inhibitors on Hyperosmoticity-Induced Secretion of IL-6 by CE Cells

CE cells were plated into 24-well plates at a density of 1 × 10^5^ cells per well. After 24 h incubation, the medium was changed. Various MAPK inhibitors (Calbiochem, San Diego, CA), including UO1026 (ERK inhibitor), SP600125 (JNK inhibitor), and SB203580 (p38 MAPK inhibitor) all at 10 *μ*M, were added to the medium. For the study of the effect of NF-*κ*B inhibitor, 5 *μ*M BAY11-7082 (Calbiochem, San Diego, CA) was added to the medium. Thirty minutes later, sodium bicarbonate was added to the medium to archive hyperosmotic medium as described above. Cells were cultured with isoosmotic or hyperosmotic medium as described above. After 24 h incubation, the conditioned media were collected and stored. IL-6 levels were determined using the human IL-6 Quantikine ELISA kit as described above. Tests were performed in triplicate.

### 2.8. Statistical Analysis

Data analysis was performed using specific software (SPSS 19.0, SPSS Inc., Chicago, IL, USA). Statistical significance was analyzed using analysis of one-way ANOVA test. *P* values less than 0.05 were considered as significant.

## 3. Results

### 3.1. MTT Assay

**Figure 1:**
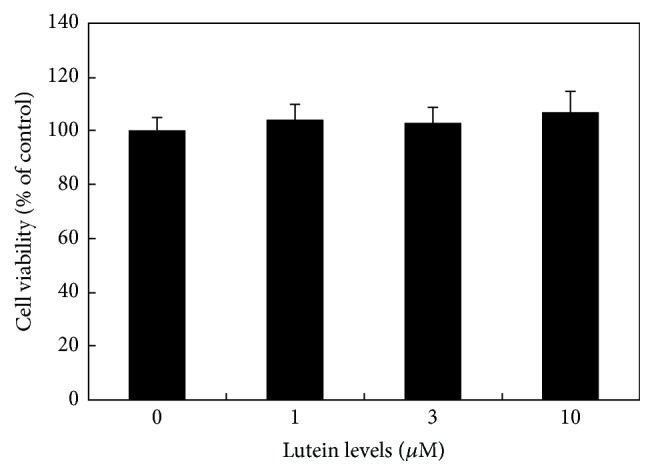
Effects of lutein on cell viability of cultured human CE cells. Cells were cultured with different levels of lutein and cell viability was measured by MTT assay. Lutein at 1, 3, and 10 *μ*M did not affect the cell viability.

Cell viability of cultured human CE cells was not affected by lutein at 1, 3, and 10 *μ*M as compared to cells cultured without lutein (*P* > 0.05) (Figure 1). Therefore, we used lutein at 1–10 *μ*M for testing its effects on hyperosmoticity-induced secretion of IL-6 and changes of various signal pathways levels in this study.

### 3.2. Effects of Lutein and Hyperosmoticity on Secretion of IL-6 by CE Cells

**Figure 2:**
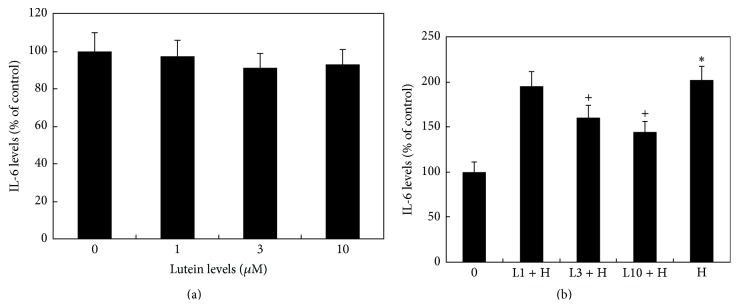
Effects of lutein and hyperosmotic medium on the secretion of IL-6 by cultured human CE cells. Cells were cultured with isoosmotic medium (0) or hyperosmotic medium (H) at 450 mOsM (b) with or without lutein at 1 *μ*M (L1), 3 *μ*M (L3), and 10 *μ*M (L10) for 24 h. IL-6 levels of conditioned medium were measured by IL-6 ELISA kit. Hyperosmotic medium caused a significant increase of IL-6 levels (^*∗*^ *P* < 0.05) as compared to cells cultured in isomer medium (0). Lutein at 3 and 10 *μ*M significantly inhibited hyperosmoticity-induced increase of IL-6 secretion (^+^ *P* < 0.05).

CE cells cultured in isoosmotic medium showed a constitutive secretion of IL-6 at 42.3 ± 4.7 ng/mL. Hyperosmotic medium (450 mOsM) caused a significant increase of IL-6 levels in the culture medium (*P* < 0.05, Figure 2).

In cells cultured with isoosmotic medium, lutein did not significantly affect IL-6 levels in the conditioned medium (*P* > 0.05, Figure 2(a)). In cells cultured with hyperosmotic medium, IL-6 levels in the conditioned medium from lutein treated cultures were dose-dependently and significantly decreased as compared to the positive controls (cells cultured with hyperosmotic medium but without lutein) (Figure 2(b)). IL-6 levels in cells treated with lutein at 3 and 10 *μ*M were significantly lower than that in the positive controls (*P* < 0.05, Figure 2(b)).

### 3.3. Effects of Lutein and Hyperosmoticity on MAPK and NF-*κ*B Levels in CE Cell

**Figure 3:**
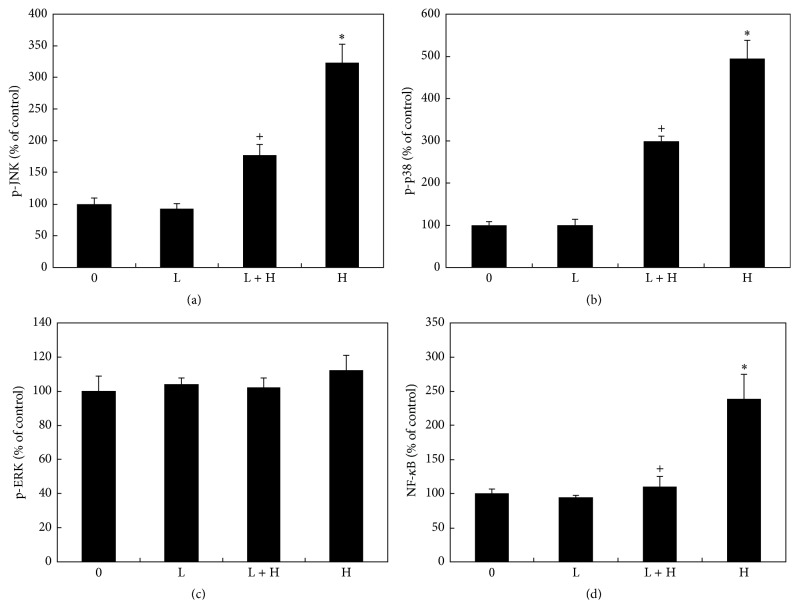
Effects of lutein and hyperosmotic medium on various signal pathways levels of cultured CE cells. Cells were cultured with isoosmotic medium (0) or hyperosmotic medium (H) at 450 mOsM with or without lutein at 10 *μ*M (L). Cells were collected and the levels of phosphorylated- (p-) JNK (a), p-p38 (b), and p-ERK (c) in cell lysates and NF-*κ*B in cell nuclear extracts (d) were measured using relevant ELISA kits, respectively. Hyperosmoticity caused a significant increase of p-p38, p-JNK, and NF-*κ*B levels (^*∗*^ *P* < 0.05) but not p-ERK levels. Lutein did not affect any pathways levels in cells cultured with isoosmotic medium. In cells cultured with hyperosmotic medium, lutein significantly reduced p-p38, p-JNK, and NF-*κ*B levels (^+^ *P* < 0.05) but not p-ERK1/2 levels as compared to cells cultured with hyperosmotic medium but without lutein.

Hyperosmoticity caused a significant increase of phosphorylated p38 MAPK and JNK1/2 in CE cells lysates (*P* < 0.05) (Figures 3(a) and 3(b)) but not p-ERK1/2 levels (Figure 3(c)). Lutein did not significantly affect p38 MAPK, JNK1/2, and ERK1/2 levels in cells cultured with isoosmotic medium (Figure 3). In cells cultured with hyperosmotic medium, lutein at 10 *μ*M significantly reduced p-p38 MAPK and p-JNK1/2 levels, but not p-ERK1/2 levels as compared to the positive controls (cells cultured with hyperosmotic medium but without lutein) (*P* < 0.05, Figure 3).

NF-*κ*B levels in cell nuclear extracts from cells treated with hyperosmotic medium were significantly greater than that of the negative controls (cells cultured with isoosmotic medium) (*P* < 0.05, Figure 3(d)). Lutein did not significantly affect NF-*κ*B levels in cells cultured with isoosmotic medium (*P* > 0.05, Figure 3(d)). In cells cultured with hyperosmotic medium, lutein at 10 *μ*M significantly reduced NF-*κ*B levels as compared to the positive controls (*P* < 0.05, Figure 3(d)).

### 3.4. Effects of MAPK and NF-*κ*B Inhibitors on Hyperosmoticity-Induced Secretion of IL-6 by CE Cells

**Figure 4:**
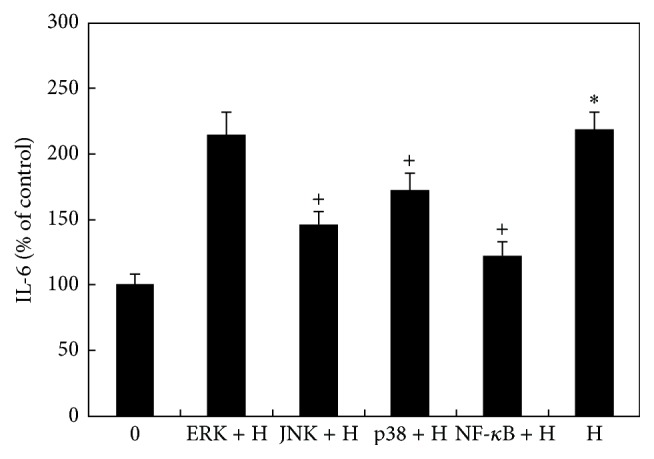
Effects of MAPK and NF-*κ*B inhibitors on hyperosmoticity-induced secretion of IL-6 by CE cells. Cells were cultured with isoosmotic medium (0) or hyperosmotic medium (H) with or without various pathway inhibitors, including UO1026 (ERK inhibitor, ERK + H); SP600125 (JNK inhibitor, JNK + H); SB203580 (p38 inhibitor, p38 + H); and BAY11-7082 (NF-*κ*B inhibitor, NF-*κ*B + H). IL-6 levels in conditioned medium were measured by using IL-6 ELISA kit. Hyperosmotic medium significantly increased IL-6 levels (^*∗*^ *P* < 0.05). JNK, p38, and NF-*κ*B inhibitors significantly inhibited hyperosmoticity-induced increase of IL-6 by CE cells (^+^ *P* < 0.05). ERK inhibitors did not affect hyperosmoticity-induced increase of IL-6.

Pretreatment of cells with SB 203580 (p38 MAPK inhibitor) or SP 600125 (JNK inhibitor) for 30 min before the cells were exposed to hyperosmotic medium significantly decreased IL-6 levels in conditioned medium as compared to the positive controls (cells cultured with hyperosmotic medium alone) (*P* < 0.05, Figure 4). Pretreatment of cells with UO1026 (ERK inhibitor) for 30 min before the cells were exposed to hyperosmotic medium did not significantly reduce IL-6 levels in conditioned medium as compared to the positive controls (*P* > 0.05, Figure 4).

In the study of the role of NF-*κ*B in hyperosmoticity-induced increase secretion of IL-6, cells pretreated with BAY11-7082 (NF-*κ*B inhibitor) significantly decreased the release of IL-6 as compared to the positive controls (Figure 4).

These results suggested that p38 MAPK, JNK, and NF-*κ*B, but not ERK, played an important role in hyperosmoticity-induced increase of IL-6 secretion by cultured CE cells.

## 4. Discussion

IL-6 is a pleiotropic cytokine that regulates multiple biological processes, including the development of the nervous and hematopoietic systems, acute-phase responses, and inflammation and immune responses [15, 16]. IL-6 is an important cytokine that amplifies immune and inflammatory responses and plays a critical role in the occurrence of autoimmune diseases. Dysregulation of the expression of IL-6 is associated with a variety of diseases, especially autoimmune diseases and inflammatory proliferative diseases, which include rheumatoid arthritis, glomerulonephritis, psoriasis, Crohn diseases, plasmacytoma, and myeloma [16].

It has been reported that tear IL-6 levels are significantly increased in dry eye patients [9–12] and the expression of IL-6 was upregulated in conjunctival tissues in Sjögren syndrome, a major cause of dry eye [13, 14]. Hyperosmoticity is the major pathological change in dry eye and plays an important role in the development of inflammation and damage of the ocular surface. Hyperosmoticity caused significant increase of IL-6 levels in various experimental animal models [17] and cultured cells [8, 18, 19], especially in cultured CE cells [8, 19]. In the present study, hyperosmoticity significantly increased IL-6 secretion of cultured human CE cells, which is consistent with previous reports.

Lutein, in addition to working as a blue light filter, is an antioxidant and also has an anti-inflammatory effect [35–39]. Lutein inhibits inflammation induced by various stimulators in vitro or in vivo [35–39]. In vitro study suggested that lutein inhibits LPS-induced expression of IL-6 in macrophages [39]. In experimental animals, supplementation of lutein decreased liver IL-6 levels in normal chicks or alcohol intoxicated rats [43, 44]. In normal individuals or patients with various diseases such as atherosclerosis and undertaken peritoneal dialysis, blood lutein or lutein/zeaxanthin levels were inversely associated with the elevation of IL-6 levels [40–42]. Supplementation of lutein decreased serum IL-6 levels in atherosclerosis patients [45]. In experimental ophthalmology, lutein inhibited LPS-induced uveitis in rats and mice [35–38] and decreased IL-6 levels in the aqueous humor [38]. In the present study, lutein significantly and dose-dependently inhibited hyperosmoticity-induced increase of the secretion of IL-6 by cultured human CE cells. This result is consistent with the results obtained from previous studies.

It has been reported that hyperosmoticity induced the expression of proinflammatory cytokines through various signal pathways. MAPK and NF-*κ*B pathways have been linked with this process [8, 18]. In the present study, hyperosmotic medium caused the increase of p-p38 MAPK, p-JNK1/2 levels, but not p-ERK1/2 levels, and also elevated NF-*κ*B levels in nuclear extracts. Also p38 MAPK, JNK1/2, and NF-*κ*B inhibitors significantly reduced hyperosmoticity-induced secretion of IL-6 by CE cells, whereas ERK1/2 inhibitor did not. These results suggested that hyperosmoticity increased IL-6 secretion of cultured CE cells by activation of p38 MAPK, JNK1/2, and NF-*κ*B pathways. This is consistent with previous reports stating that hyperosmoticity increased IL-6 secretion by cultured CE cells via p38 MAPK, JNK1/2, and NF-*κ*B pathways [8].

It has been reported that MAPK and NF-*κ*B pathways play a role in proinflammatory factors-induced expression of IL-6 in different cells or tissues [16, 46–49]. In the present study, lutein significantly inhibited hyperosmoticity-induced elevation of IL-6 expression, and this effect was associated with the activation of p38 MAPK, JNK1/2, and NF-*κ*B pathways, suggesting that p38 MAPK, JNK1/2, NF-*κ*B are the upstream of the expression of IL-6. This is consistent with the fact that LPS stimulated IL-6 expression in macrophages and hyperosmoticity-induced expression of IL-6 in cultured CE cells through p38 MAPK, JNK1/2, and NF-*κ*B pathways [8, 50].

Dry eye is a very common ocular disorder and characterized by deficiency of tear associated with inflammation and damage of ocular surface. There are numerous therapeutic procedures available for the treatment of dry eye, but all have their limitations [3–7]. The most common treatment for the dry eye is the use of topical tear substitutes. Tear substitutes provide palliative relief to eye irritation in dry eye patients. However, this effect is temporary and symptom-relief only [6]. Topical corticosteroids are effective in controlling inflammation and decreasing the signs and symptoms associated with dry eye and Sjögren syndrome. However, the possible complications associated with long-term usage, such as cataracts, glaucoma, and infection, limit their use to short-term or pulse therapy only [5, 6]. Topical cyclosporine (an immunosuppressive and anti-inflammatory drug) alleviates the signs and symptoms of dry eye. However, some patients experience bothersome adverse effects (e.g., burning or irritation) and not all of the patients with dry eye show a consistent therapeutic response to this treatment [3, 6]. Topical autologous serum also improves dry eye symptoms and signs. The limitations of using autologous serum include the nuisance of preparation, the need to refrigerate the drops, and the potential risk of infection if contamination of the solution occurs [5]. Therefore, novel efficient therapies with few or no side-effects for the management of dry eye patients are required. Lutein is a safe dietary supplement and has been used for the treatment of AMD and other ocular diseases for a long time [31–34]. The promising results of lutein on the inhibition of hyperosmoticity-induced elevation of IL-6 expression obtained from the present study support further investigation of the use of lutein in the treatment of dry eye.

In conclusion, this study suggested that lutein inhibited hyperosmoticity-induced elevation of secretion of IL-6 by cultured CE cells through the inhibition of p38 MAPK, JNK1/2, and NF-*κ*B pathways. Lutein has been used for the treatment of various eye diseases without untoward effects. Therefore, lutein may be a promising agent to be explored for the treatment of dry eye.
